# Comparative genome characterization of *Echinicola marina* sp. nov., isolated from deep-sea sediment provide insight into carotenoid biosynthetic gene cluster evolution

**DOI:** 10.1038/s41598-021-03683-0

**Published:** 2021-12-17

**Authors:** Yu Pang, Mengru Chen, Wei Lu, Ming Chen, Yongliang Yan, Min Lin, Wei Zhang, Zhengfu Zhou

**Affiliations:** Biotechnology Research Institute, Chinese Academy of Agricultural Sciences, Beijing, 100081 China

**Keywords:** Evolution, Microbiology

## Abstract

*Echinicola*, carotenoid-pigmented bacteria, are isolated from various hypersaline environments. Carotenoid accumulation in response to salt stress can stabilize the cell membrane in order to survive. A pink-colored strain SCS 3–6 was isolated from the deep-sea sediment of the South China Sea. Growth was found to occur at 10–45 °C. The strain could tolerate 10% (w/v) NaCl concentration and grow at pH 5–9. The complete genome of SCS 3–6 comprises 5053 putative genes with a total 5,693,670 bp and an average G + C content of 40.11 mol%. The 16S rRNA gene sequence analysis indicated that strain SCS 3–6 was affiliated with the genus *Echinicola,* with the closely strains were *Echinicola arenosa* CAU 1574^T^ (98.29%)and *Echinicola shivajiensis* AK12^T^ (97.98%). For *Echinicola* species with available genome sequences, pairwise comparisons for average nucleotide identity (ANI) and in silico DNA-DNA hybridization (DDH) revealed ANIb values from 70.77 to 74.71%, ANIm values from 82.72 to 88.88%, and DDH values from 18.00 to 23.40%. To identify their genomic features, we compared their genomes with those of other *Echinicola* species. Phylogenetic analysis showed that strain SCS 3–6 formed a monophyletic clade. Genomic analysis revealed that strain SCS 3–6 possessed a complete synthetic pathway of carotenoid and speculated that the production was astaxanthin. Based on phenotypic and genotypic analyses in this study, strain SCS 3–6 is considered to represent a novel species of the genus *Echinicola* for which the name *Echinicola marina* sp. nov. is proposed. The type strain is SCS 3-6^T^ (= GDMCC 1.2220^T^ = JCM 34403^T^).

## Introduction

Carotenoids are yellow, orange or red isoprene pigments from a wide range of sources, which can be synthesized by photosynthetic organisms (plants, cyanobacteria, anoxygenic phototrophic bacteria and aerobic anxoygenic phototrophic bacteria), non-photosynthetic microorganisms^1,2^. There are more than 600 carotenoids found naturally. Carotenoids have high commercial application value. At present, the carotenoids commonly used as animal feed and food additives on the market mainly include lutein, β-carotenenes, lycopene and astaxanthin^3^. Astaxanthin (3,3’-dihydroxy-β,β b-caroten-4,4’-dione) is a xanthophyll carotenoid which is a secondary metabolite responsible for a red fat-soluble pigment^4^. It is produced predominantly by marine microorganisms and animals^5,6^. Astaxanthin has several essential biological activities such as powerful antioxidant, anti-inflammatory, and antiapoptotic activities^7,8^. Natural astaxanthin is Generally Recognized as Safe (GRAS)^9^. It is widely used in the food, animal feed, nutraceutical and pharmaceutical industries.

The genus *Echinicola* was first described by Nedashkovskaya et al.^10^. The member of the genus *Echinicola* are characterized as Gram-negative, aerobic or facultatively anaerobic, rod-shaped and pigmented bacteria^10^. The genus is affiliated with the family *Cyclobacteriaceae* belonging to the class *Cytophaia* of the phylum *Bacteroidetes*. At the time of writing, the genus *Echinicola* accommodates 9 species with validly published names and 3 published but not valid species: *Echinicola pacifica*^10^, *Echinicola vietnamensis*^11^, *Echinicola jeungdonensis*^12^, *Echinicola roses*^13^, *Echinicola sediminis*^14^, *Echinicola strongylocentroti*^15^, *Echinicola soli*^16^, *Echinicola arenosa*^17^, *Echinicola salinicaeni*^18^ and ‘*Echinicola shivajiensis*’^19^. These *Echinicola* type strains were isolated from various hypersaline environments, including sea urchin^10,15^, sea water^11,13,17^, saline soil^16,18^, solar saltern^12^, coastal sediment^14^ and brackish water^19^.

Marine *Bacteroidetes* are commonly assumed to be specialized in degrading polysaccharides^20^ due to a great number and diversity of carbohydrate-active enzymes (CAZymes) in their genomes^21^. *Echinicola rosea* JL3085^T^ genome harbors multi-gene polysaccharide utilization loci (PUL) systems involved in the degradation of pectin, xylan and arabinogalactan^22^. And a xylanase gene (*xynT*) from *Echinicila rosea* JL3085^T^ was cloned and recombinantly expressed in *Escherichia coli* BL21^23^. The O-specific polysaccharides are isolated from the lipopolysaccharide of *Echinicola vietnamensis* KMM 6221^T^ and *Echinicola pacifica* KMM 6172^T^ that reveals the polysaccharide is built up of branched tetrasaccharide repeating units^24,25^. Therefore, it is suggested that *Echinicola* strains play an important role in the carbon cycle in the marine environment.

Strain SCS 3–6, a pink-pigmented strain was isolated from deep sea sediment of the South China Sea and found to belong to the genus *Echinicola* based on 16S rRNA gene sequences and its phenotypic characteristics. And the whole genome sequences were obtained for the newly strain. In addition, whole genome sequences of some type strains of Echinicola species are available in GenBank database, the genome sequences have not been used previously for taxonomic analysis or subjected to comparative genomic studies. Therefore, we used the available genome sequence of *Echinicola* species to investigate the taxonomic status of the newly isolated strains with the genus *Echinicola* and to identify the differences by genotypic characteristics. With genotypic analysis and the examination of its carotenoid production ability, strain SCS 3–6 is an astaxanthin-producing strain.

## Materials and methods

### Sampling sites, enrichment and isolation

Strain SCS 3–6 was isolated from deep-sea sediment sample collected from the South China Sea (depth of 1700 m, E 117°56.2877′, N 20°59.8047′). In enrichment experiments for isolating strain SCS 3–6, 1 g of sediment sample was enriched in 50 ml marine agar (Difco) for 72 h at 28 °C, 150 rpm. Then, 200 μl of enriched solution was transferred to fresh medium and was cultured at 28 °C, 150 rpm for 72 h. And this routine culturing was repeated three times. After, the supernatants of the enriched sample were serially diluted (10^–5^ to 10^–7^) with PBS buffer (KH_2_PO_4_ 0.2 g, Na_2_HPO_4_·12H_2_O 2.9 g, NaCl 8 g, KCl 0.2 g, pH 7.0). 100 μl of each diluted sample was spread on marine agar plates and incubated at 28 °C for 48 h.

### Morphological, physiological, and biochemical analysis

The morphological characteristics of the strains were investigated after 24 h of incubation on marine agar. The Gram reaction was examined according to Buck’s method^26^. Cell morphology was investigated using scanning electron microscope (SU8010, Hitachi, Japan) and transmission electron microscope (H-7650, Hitachi, Tokyo, Japan). Growth was observed at various temperatures (4, 15, 20, 25, 28, 30, 33, 37, 40, 45, and 50 °C) on marine agar. Tolerance to different NaCl concentrations (0–10%, in increments of 1%, w/v, NaCl) and pH range (pH 4.0–11.0, at intervals of 1 unit) were performed at 28 °C, for 7 days. Anaerobic growth was tested in an MGCAnaeroPouch-Anaero (Mitsubishi, Tokyo, Japan) at 28 °C for 7 days on marine agar plates. Catalase and oxidase activities were investigated in 3% (v/v) H_2_O_2_ and using commercial strips (Huankai, Guangzhou, China) according to the manufacturer’s instruction, respectively. Additional enzyme activities and carbon source utilization assays were examined by using API 20NE, API ZYM (bioMerieux, Marcy-l′Etoile, French) and Biolog plates kits (Hayward, CA, USA), respectively, following the manufacturer’s instruction.

### Chemotaxonomic analysis

For analysis of the chemotaxonomic features of strain SCS 3–6, a series of experiments^27^ were carried out to determine the content of the respiratory quinones, polar lipids, and fatty acids of closely related type strains (*Echinicola shivajiensis* JCM 17847^T^ and *Echinicola sediminis* KCTC 52495^T^) and SCS 3–6 with cell biomass obtained from cultures grown in marine agar (Difco) for 2 days at 28 °C. Respiratory quinones were extracted from freeze-dried cells (100 mg) with chloroform/methanol (2:1) and analyzed via the HPLC system^28^. Polar lipids were extracted by using a chloroform/methanol/water system^29^ and separated by two-dimensional TLC. The plate dotted with the sample was subjected to two-dimensional development, with the first solvent of chloroform/methanol/water (65:25:4, by vol.) followed by the second solvent of chloroform/methanol/acetic acid/water (85:12:15:4, by vol.). The polar lipids were identified by spraying with phosphomolybdate, ninhydrin, Dragendorff’s reagents, molybdenum blue, 1-methylnaphthol, respectively. For cellular fatty acid analysis, fatty acids were saponified, methylated, and extracted according to the Microbial Identification System (MIDI) protocol. The fatty acid methyl eaters, analysed with a gas chromatograph (7890, Hewlett Packard), were identified by the Microbial Identification software package, based on the Sherlock Aerobic Bacterial Database (TSBA6)^30^.

### Sequencing and phylogenetic analyses

Amplification of the 16S rRNA gene was performed by PCR with two primers: 27F and 1492R^31^. The amplified gene was ligated into the pJET1.2/Blunt Vector (Thermo Scientific, Waltham, MA, USA) and sequenced by Sangon Biotech (Shanghai, China). The 16S rRNA gene sequences used EzBioCloud’s Identify services^32^ to get sequences informations. The phylogenetic tree was constructed using the ClustalW algorithm from the MEGA 11^33^ software package using the neighbor-joining (NJ) and maximun likelihood (ML) methods followed by bootstrap analysis with 1000 bootstrap replications^34^.

### Genome DNA sequencing, de novo assembly, annotation and genomes comparison

For genomic DNA extraction and sequencing, strain SCS 3–6 was inoculated from glycerol stocks in marine agar, and grown for 24 h at 28 °C, 200 rpm. Then, bacteria were washed in 1 × PBS and collected by centrifugation at 5000 rpm for 10 min at 4 °C. The genome of SCS 3–6 was extracted and sequence by Majorbio Bio-pharm Technology Co., Ltd (Shanghai, Chain) on PacBio and illumina Hiseq × 10 platform. A high-quality data set with a corresponding sequencing depth of 100-fold was generated. The scan map of the bacterial genome is created using SOAPdenovo2^35^ and the complete map of the bacterial genome is assembled using Canu and SPAdes^36^. Glimmer and GeneMarkS^37^ were used to predict coding sequences (CDS) and plasmid genes, respectively. tRNA and rRNA were predicted using tRNAscan-SE v2.0 and Barrnap, respectively. Function annotation of SCS 3–6 was obtained from Non-Redundant Protein (NR), Swiss-Prot, Pfam, Clusters of Orthologous Group (COG), Gene Ontology and Kyoto Encyclopedia of Genes databases using BLASTp and the same BLAST thresholds^38^. Additionally, the CAZymes were identified, classified and annotated using CAZy database^39^. The gene clusters of secondary metabolites were identified by antiSMASH program^40^.

The average nucleotide identity (ANI) was calculated using the BLAST (ANIb) and MUMmer (ANIm) algorithms (http://jspecies.ribohost.com/jspeciesws/) ^41^ DNA–DNA hybridization (DDH) was calculated according to the method described by Meier-Kolthoff et al.^42^. The BPGA pipeline^43^ was used to perform model extrapolations of the *Echinicola* pangenome/core genome by applying default parameters.

### The assay of its carotenoid production ability

The strains were inoculated into 100 mL marine agar, cultured at 28 °C for 72 h, centrifuged, and the bacteria were collected. Add 3 mL sterile water to the tube and wash the bacteria. Transfer the bacteria to a 15 mL centrifuge tube. Centrifuge at 4 °C, 8000×*g* for 10 min, then discard supernatant. The bacteria were resuspended with 2 mL acetone solution and extracted by shock for 10 min. After centrifugation at 8000×*g* for 10 min, the supernatant extract was transferred to a new tube. 2 mL ethyl acetate solution was added to the original tube, and the extraction was continued with oscillation for 10 min. After centrifugation at 8000×*g* for 10 min, the supernatant extract was mixed with the previous extract, and 3 mL sterile water was added to it, and the mixture was shaken and mixed. Centrifuge 8000×*g* for 10 min to delaminate the liquid and draw the upper liquid into a new EP tube. The ethyl acetate was dried to obtain powder and was redissolved with methanol. Over 0.22 µm filter membrane, used for High-pressure liquid chromatography (Shimadzu HPLC LC-20AT) analysis and detection. The tests were by C18 column with a mobile phase of methanol–acetonitrile–water (volume ratio being 80:15:5). UV detection was performed at 478 nm.

### Ethical approval

This article does not contain any studies with human participants or animals performed by any of the authors.

## Results and discussion

### Phylogenetic characteristics

**Figure 1:**
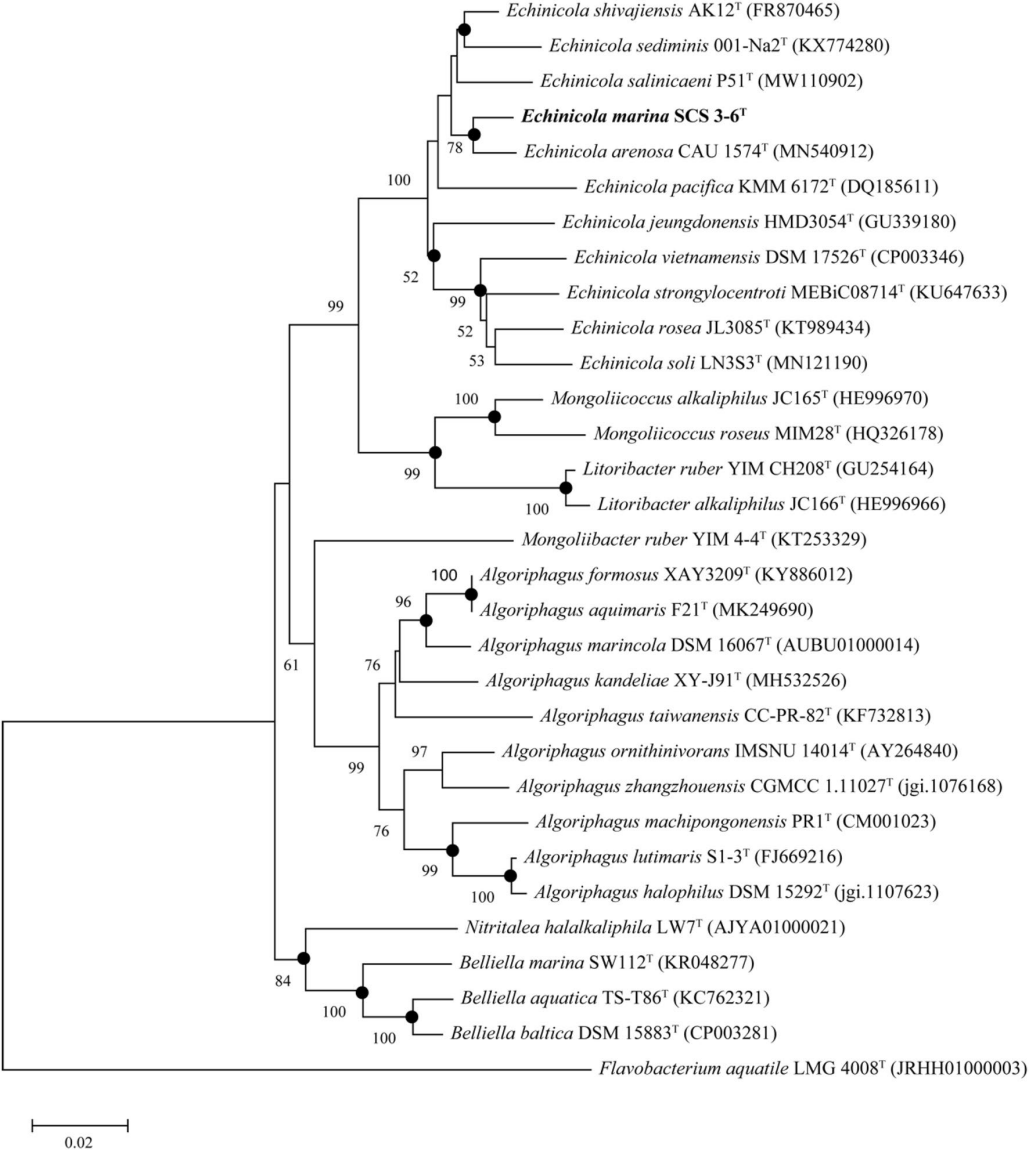
Phylogenetic tree constructed with 16S rRNA gene by the NJ algorithm. The tree was rooted using *Flavobacterium aquatike* LMG4008^T^ as the outgroup. Bootstrap values (> T50%) are shown at the nodes. Closed circles (·) represent branches recovered in maximum likelihood (ML) tree. The phylogenetic tree was constructed using the ClustalW algorithm from the MEGA 11 software (MEGA 11: https://www.megasoftware.net).

The search of 16S rRNA gene sequence against the EzTaxon database revealed that strain SCS 3–6 belongs to *Bacteroidetes* phylum. The nearly complete 16S rRNA gene sequence (1518 bp) of strain SCS 3–6 was compared with other strains with the top 30 sequence similarity using phylogenetic tree analysis. Strain SCS 3–6 was 98.29% similar to *Echinicola arenosa* CAU 1574^T^, 97.98% with *Echinicola shivajiensis* AK12^T^, 96.97% with *Echinicola sediminis* 001-Na2^T^, and 96.22% with *Echinicola jeungdonensis* HMD3054^T^. The other species of the genus *Echinicola* showed < 96% sequence similarities with strain SCS 3–6. The NJ phylogenetic tree revealed strain SCS 3–6 clustered with members of the genus *Echinicola* and formed a monophyletic clade with *Echinicola arenosa* CAU 1574^T^ (Fig. 1).

### Phenotypic properties

**Figure 2:**
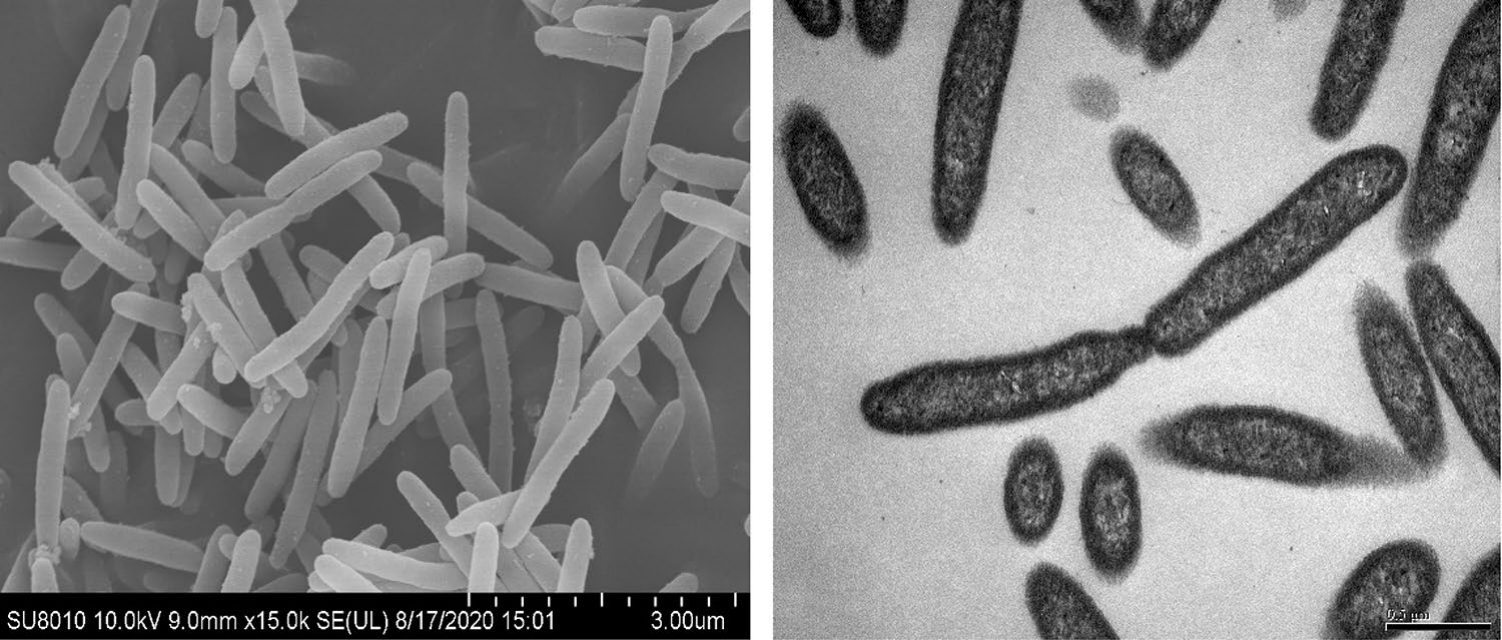
Morphology of *Echinicola marina* sp.nov. Scanning electron microscope (SEM) images of strain SCS 3–6 (left) and transmission electron microscope (TEM) images of strain SCS 3–6 (right).

Cells of strain SCS 3–6 were Gram-stain negative, non-motile by gliding, and rod-shaped (0.2–0.3 μm in width and 1.5–2.0 μm in length) (Fig. 2). Colonies of this strain were circular, convex and pink pigmented after 2–3 days of incubation 30 °C on marine agar plates. Strain SCS 3–6 were facultative anaerobes. The strain SCS 3–6 was capable of growth at temperatures between 10 and 45 °C and the strain grew well at pH values between 5.0 and 9.0. The strain SCS 3–6 was tolerant to 10% (w/v) NaCl.

The novel isolate can use various carbohydrates for growth. The following carbohydrates were utilized: d-maltose, d-trehalose, d-cellobiose, gentiobiose, sucrose, d-turanose, α-d-lactose, d-melibiose, d-salicin, *N*-acetyl neuraminic, α-d-glucose, d-mannose, d-fructose and d-galactose. Catalase and oxidase reactions were both positive. Strain SCS 3–6 was positive for the hydrolysis of gelatin and casein, but negative for starch and tween 80. Biochemical characteristics that differ between strain SCS 3–6, *Echinicola shivajiensis* AK12^T^ and *Echinicola sediminis* 001-Na2^T^ are shown in Table 1.

**Table 1:** Differential physiological characteristics between strain SCS 3–6 and closely related type strains.

Characteristic	1	2	3
Production of catalase	+	+	+
Oxidase	+	+	+
Nitrate reductase	−	−	−
Gelatin hydrolysis	+	+	−
Casein hydrolysis	+	+	−
**Oxidation of (Biolog GENIII)**			
Sucrose	+	+	+
Dextrin	−	w	−
d-Maltose	+	+	+
d-Trehalose	+	+	+
d-Cellobiose	+	+	+
Stachyose	w	+	+
d-Raffinose	w	+	+
d-Salicin	+	+	w
α-d-Glucose	+	+	+
d-Mannose	+	+	+
d-Fructose	+	+	+
d-Galactose	+	+	+
l-Rhamnose	w	w	+
Sodium lactate	w	+	+
d-Mannitol	−	w	−
Glycerol	−	−	w
Gelatin	−	w	−
l-Aspartic acid	w	+	w
l-Arginine	−	−	w
l-Lactic acid	−	−	w

1, *E. marina* SCS 3–6; 2, *E. shivajiensis* AK12^T^; 3, E. *sediminis* 001-Na2^T^.

All data are from this study. + , Positive; -, negative; w, weakly positive.

**Figure 3:**
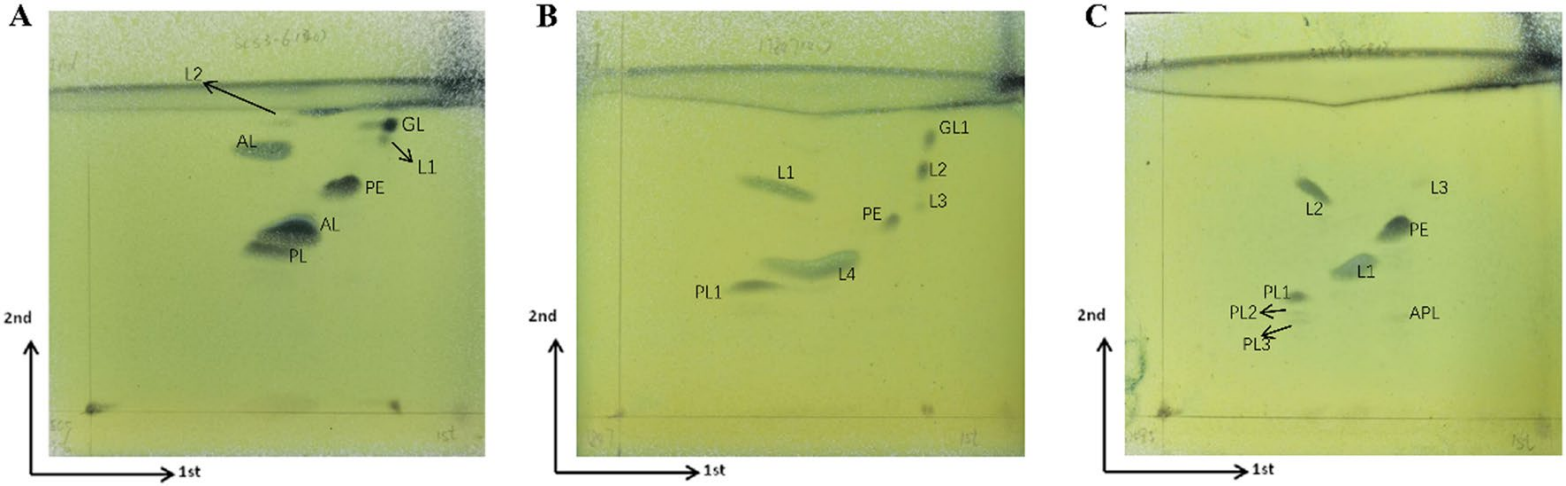
The fatty acid profiles of strain SCS 3–6 and its relatives. **(A)*** E. marina* SCS 3–6; **(B)** *E. shivajiensis* JCM 17847^T^; **(C)** *E. sediminis* KCTC 52495^T^. *PE* phosphatidylethanolamine, *PL* unidentified phospholipid, *GL* unidentified glycolipid, *AL* unidentified aminolipid, *L* unidentified lipid, *APL* unidentified aminophospholipid.

The major cellular fatty acids were iso-C_15:0_ and Summed Feature 3 (C_16:1_ ω7c and/or C_16:1_ ω6c) (Supplementary Table S1). The major isoprenoid quinone was MK-7. The polar lipids were phosphatidylethanolamine, an unidentified phospholipid, an unidentified glycolipid, two unidentified aminolipids, two unidentified lipids (Fig. 3).

### General genome features and genetic relatedness

**Figure 4:**
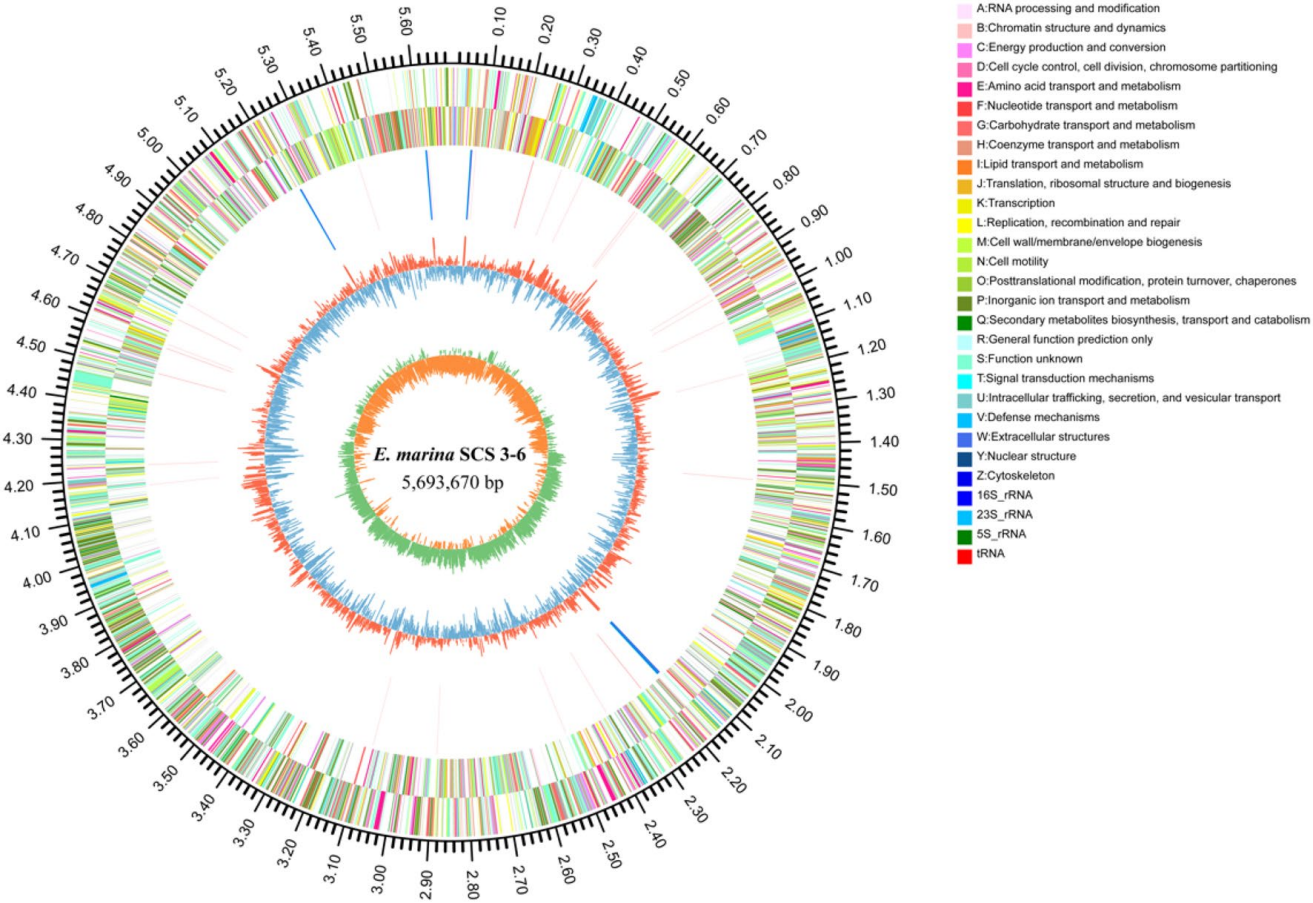
Circular genome map of *Echinicola marina* SCS 3–6. The circular map was generated using Circos and contain seven circles (http://www.circos.ca). Marked information is displayed from the outer circle to innermost, as follows: genomes size, CDSs on the forward stand, CDSs on the reverse stand, the distribution of rRNAs and tRNAs, GC content, GC skew.

The complete genome of strain SCS 3–6 using the circus program^44^ to visualize contained a single circular chromosome of 5,693,670 bp with a guanine-cytosine (GC) content of 40.11 mol% (Fig. 4). No plasmids were present in the strain SCS 3–6 genome. In total, 5053 coding sequence regions, 41 tRNA genes, and 5 sets of rRNA genes (5S,16S, and 23S rRNA genes) were respectively predicted (Supplementary Table S2). The GC content of strain SCS 3–6 was the lowest among all strains of current *Echinicola* strains.

ANIb, ANIm, dDDH, and OrthoANI values were calculated to identify the genomic similarities of strain SCS 3–6 to the six strains of *Echinicola* species with available genome sequences. ANIb, ANIm, and dDDH values are presented in Table 2 and OrthoANI values are shown in Supplementary Fig. S1. The ANIb between SCS 3–6 and *Echinicola pacifica* DSM 19836^T^ was 70.77 (70.66), and that between SCS 3–6 and *Echinicola arenose* CAU 1574^T^ was 74.56 (74.71), substantially less than the 95% to 96% threshold for species demarcation proposed previously^45^. The values for ANIm among the strain SCS 3–6 and other *Echinicola* species were from 82.72% to 88.88%. Using dDDH analysis, strain SCS 3–6 exhibited values ranging from 18.0% with *Echinicola pacifica* DSM 19836^T^ to 23.4% with *Echinicola rosea* JL3085^T^ and *Echinicola vietnamensis* DSM 17526^T^ (Table 2). A phylogenetic tree of seven *Echinicola* species based on the OrthoANI algorithm showed the analyzed *Echinicola* species could be divided into three clades (Supplementary Fig. S1). Strain SCS 3–6 formed a clade with *Echinicola aremosa* CAU 1574^T^. And the strain SCS 3–6 showed OrthoANI ranging from 71.41% with *Echinicola pacifica* DSM 19836^T^ to 75.20% with *Echinicola aremosa* CAU 1574^T^. These values are lower than the 70% threshold for dDDH and the 95–96% threshold for ANI used for delineating prokaryotic species, thus confirming that this strain represents a new species.

**Table 2:** ANIb, ANIm, and dDDH values between pairs of type strains of *Echinicola* species.

		1	2	3	4	5	6	7
**ANIb**								
1	SCS 3–6	*	70.77	72.40	72.53	73.71	73.84	74.71
2	*Echinicola pacifica* DSM 19836^T^	70.66	*	71.35	71.20	71.27	71.20	70.59
3	*Echinicola soli* LN3S3^T^	72.52	71.43	*	81.66	79.80	82.71	72.80
4	*Echinicola strongylocentroti* MEBiC08714^T^	72.51	71.20	81.50	*	78.57	83.01	72.82
5	*Echinicola vietnamensis* DSM 17526^T^	73.75	71.30	79.71	78.64	*	80.92	72.58
6	*Echinicola rosea* JL3085^T^	73.65	71.21	82.47	82.80	80.81	*	72.63
7	*Echinicola arenose* CAU 1574^T^	74.56	70.66	72.64	72.66	72.43	72.64	*
**ANIm**								
1	SCS 3–6	*	82.70	83.05	84.34	88.88	88.01	84.35
2	*Echinicola pacifica* DSM 19836^T^	82.72	*	82.51	82.64	82.87	82.74	82.99
3	*Echinicola soli* LN3S3^T^	83.08	82.52	*	85.82	84.24	85.47	82.68
4	*Echinicola strongylocentroti* MEBiC08714^T^	84.30	82.64	85.82	*	84.41	87.24	83.09
5	*Echinicola vietnamensis* DSM 17526^T^	88.88	82.88	84.24	84.41	*	85.79	83.04
6	*Echinicola rosea* JL3085^T^	88.01	82.76	85.47	87.24	85.79	*	83.67
7	*Echinicola arenose* CAU 1574^T^	84.36	83.01	82.67	83.06	83.03	83.65	*
**dDDH**								
1	SCS 3–6	*						
2	*Echinicola pacifica* DSM 19836^T^	18.00%	*					
3	*Echinicola rosea* JL3085^T^	23.40%	18.50%	*				
4	*Echinicola soli* LN3S3^T^	19.30%	18.60%	26.70%	*			
5	*Echinicola strongylocentroti* MEBiC08714^T^	20.00%	18.40%	28.50%	26.00%	*		
6	*Echinicola vietnamensis* DSM 17526^T^	23.40%	18.60%	25.30%	23.90%	23.00%	*	
7	*Echinicola arenose* CAU 1574^T^	19.70%	17.70%	19.40%	18.80%	19.30%	18.90%	*

* represents the same strain.

### Core genome and pangenome of *Echinicola*

**Figure 5:**
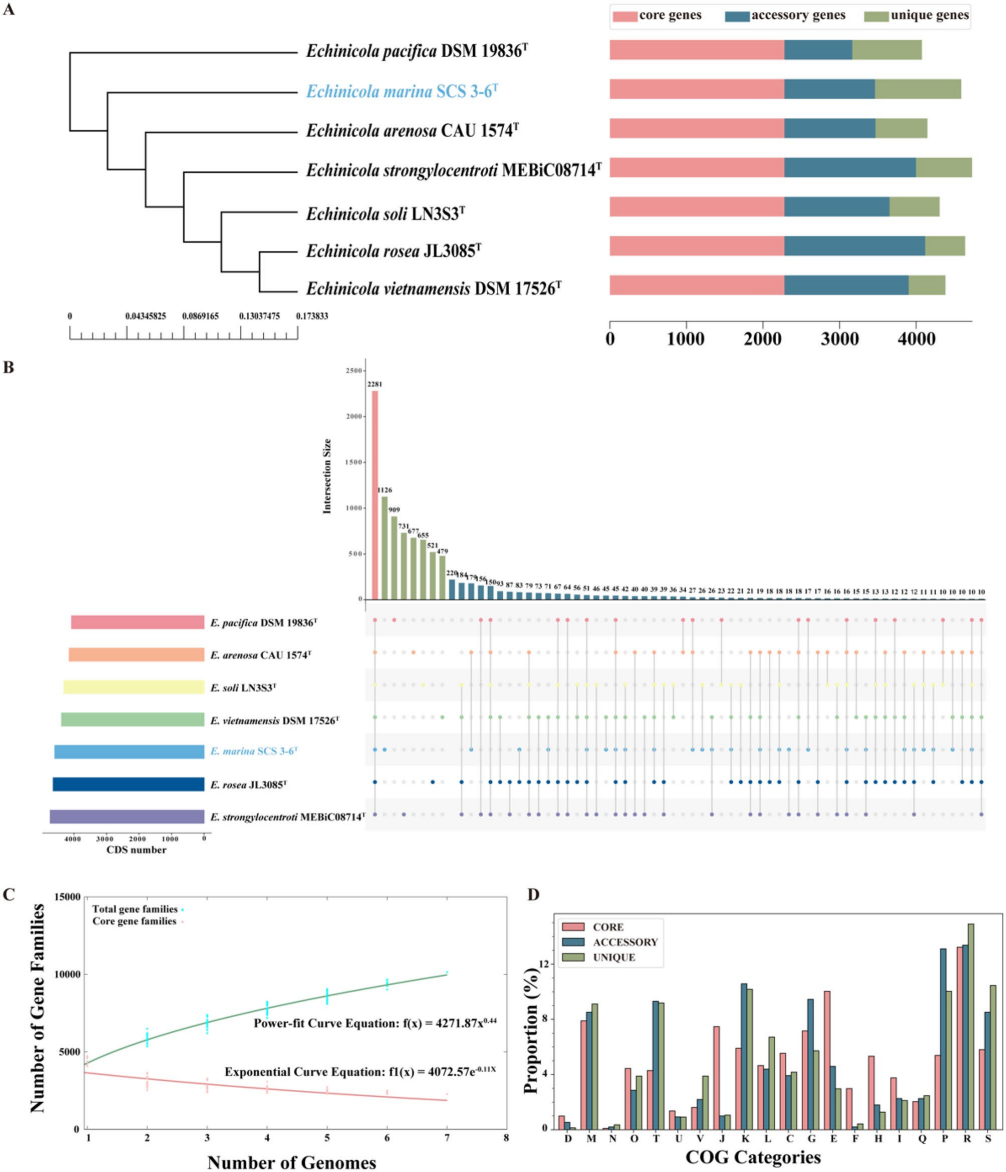
Pan-genome analysis of *Echinicola marina* strains. **(A)** Neighbor-joining tree derived from 2281 core orthologous proteins. The tree showing the phylogenetic position of strain SCS 3–6 and closely related type strains (left) and the distribution of core genes, accessory genes and unique genes of above the strains (right). **(B)** Visualization of core and accessory genomes of *Echinicola*. **(C)** Mathematical modeling of the pangenome and core genome of *Echinicola*. **(D)** Bar chart showing functional proportions (based on COG categories) of different parts of the *Echinicola* pangenome (i.e., core, accessory, unique). The extrapolations of the Echinicola pangenome/core genome were generated using BPGA pipeline (BPGA: http://www.iicb.res.in/bpga/index.html).

The genome of strain SCS 3–6 was compared to the available genomes of other *Echinicola* strains. The core genome sequences of individual strain were calculated. The resulting phylogenetic analysis indicated that strain SCS 3–6 formed a monophyletic clade (Fig. 5A). The pangenome of the 7 *Echinicola* strains possessed 10,145 gene families, while the core genome possessed 2281 gene families accounting for only 24.7% of the pangenome. The number of unique proteins in strain SCS 3–6 was 1216; specific unique protein in other *Echinicola* varied from 479 (*E. vietnamensis*) to 909 (*E. pacifica*) (Fig. 5B). Core and pangenome analyses of the 7 *Echinicola* genomes reveal an “open” pangenome fitted into a power-law regression function [f(X) = 4271.87X^0.44^], while the core genome was fitted into an exponential regression [f(X) = 4072.57e^−0.11X^] (Fig. 5C). The open pangenomes suggested that species have undergone considerable gene exchanging to extend their functional profiles^46^.

Functional COG annotation revealed that the core genome had a higher proportion of genes classified in COG categories J (translation, ribosomal structure, and biogenesis), E (amino acid transport and metabolism), F (nucleotide transport and metabolism), H (coenzyme transport and metabolism), and I (lipid transport and metabolism), which all were associated with basic biological functions and sustained life activities. The accessory genome and strain-specific genes were biased toward COG categories T (signal transduction mechanisms), K (transcription), and P (inorganic ion transport and metabolism) (Fig. 5D), which all were about informatics metabolism and were probably related to the adaption of *Echinicola* to various extreme habitats such as saline environments to accommodate their lifestyles (Supplementary File).

### Polysaccharide utilization in the genome of *E. marina*

**Figure 6:**
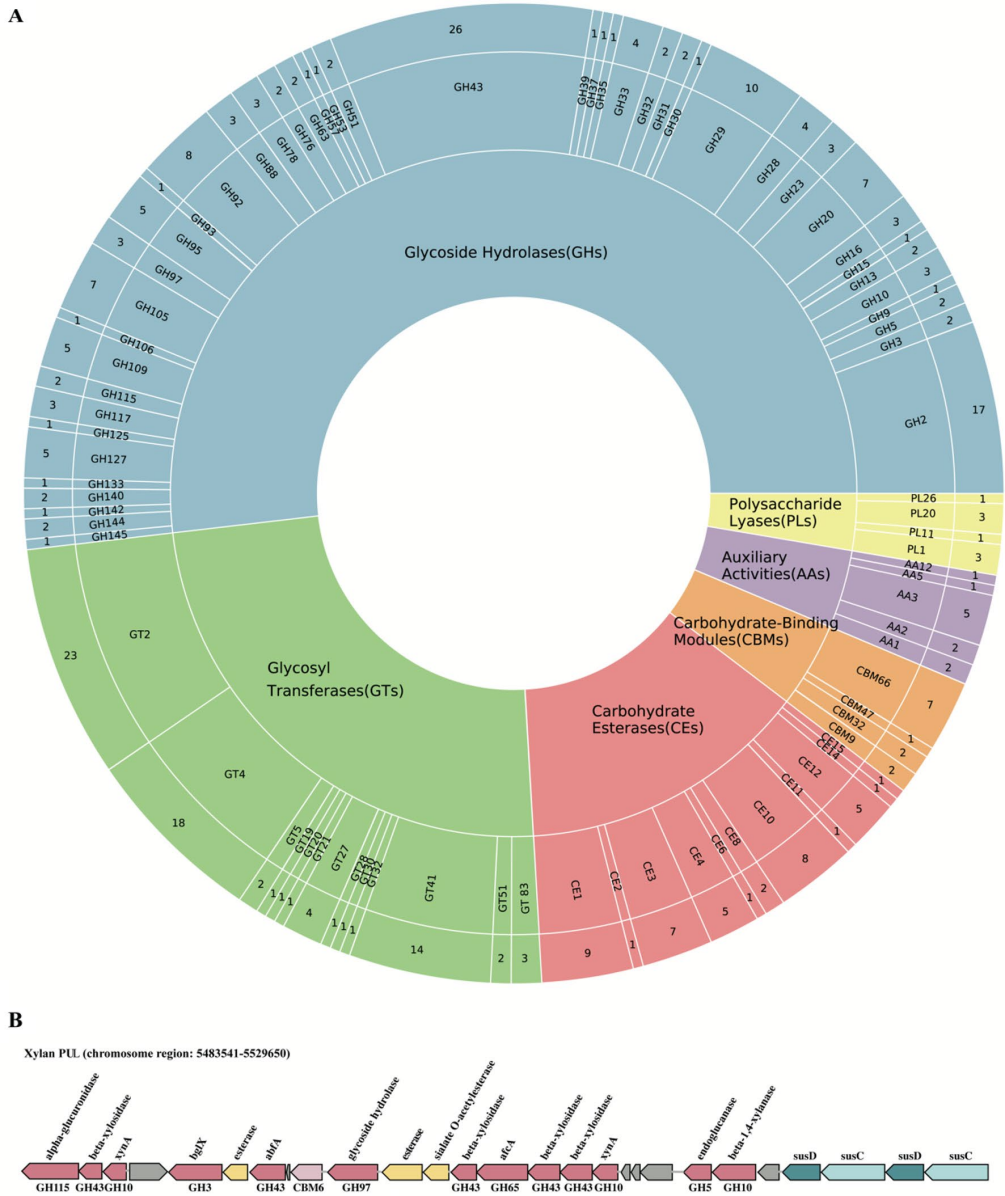
Polysaccharide utilization prediction of *Echinicola marina* strain. **(A)** CAZymes distribution in SCS 3–6 genome. Different colors represent different classes of CAZymes found in the genome. The representation from the inner to outer rings are, CAZyme classes, CAZyme families, and the number of genes identified in each family, respectively. **(B)** Genetic organization of the predicted xylan PUL in *E. marina* SCS 3–6.

Marine *Bacteroidetes* are well known for their functional specialization on the decomposotion of polysaccharides which results from a great number of carbohydrate-active enzymes^47^. Therefore, marine bacteria may provide the most common CAZymes resources for polysaccharides degradation. The genome of SCS 3–6 harbors 299 CAZymes (Fig. 6A), including Glycoside Hydrolase (GH), Glycosyl Transferase (GT), Carbohydrate Esterase (CE), Auxiliary Activity (AA), Carbohydrate-Binding Module (CBM), and Polysaccharide Lyase (PL). The largest family found in the SCS 3–6 genome was the GH, which encoded 155 genes (Fig. 6A). The putative genes encoding GHs belonged to 43 different families with gene numbers ranging between 1 and 26, in which GH43 genes was highest. Generally, GH enzymes have great potential to hydrolyze complex carbohydrates and they are considered the key enzymes involved in carbohydrate metabolism. And GHs can degrade the most abundant biomasses. A deeper analysis toward differentiation of GHs revealed SCS 3–6 can degrade xylan. GH43 are classified based on their mode of action and substrate preference into xylanases, xylosidases, arabinofuranosidase, arabinosidase and others in SCS 3–6 genome, indicating that SCS 3–6 could have the capacity of xylan utilization. In addition, numerous genes assigned to other GH families involved in the degradation of xylan were detected including three xylanases from GH10, one xylanase from GH30, two xylosidases from GH31 and two arabinofuranosidases from GH51. Additionally, two GH115 genes predicted in SCS 3–6 can cleave glucuronic acid side chains from native xylans.

PULs were manually detected based on the presence of CAZyme clusters. Some xylanolytic enzymes of *E. marina* SCS 3–6 were located on the multi-gene polysaccharide utilization loci (PUL), including genes that encode *xynA*-encoding xylanases (GH10), beta-xylosidase (GH43), alpha-glucuronidase (GH115) and one carbohydrate-binding modules (CBM6) (Fig. 6B). The xylan PUL contained genes the encode a susC–susD system and was similar to that in *E. rosea* JT3085^T^^22^. Xylans are heteropolymers containing xylose, arabinose, glucuronic acid, galactose and other residues. Therefore, the xylan PUL included *abfA*-encoding alpha-N-arabinofurnosidases (GH43) and afcA-encoding alpha-l-fucosidase (GH65). And some auxiliary activity enzymes (two esterase genes and one gene-encoding sialate *O*-acetylesterase) were involved in xylan degradation.

### Carotenoids biosynthesis capability of *E. marina*

**Figure 7:**
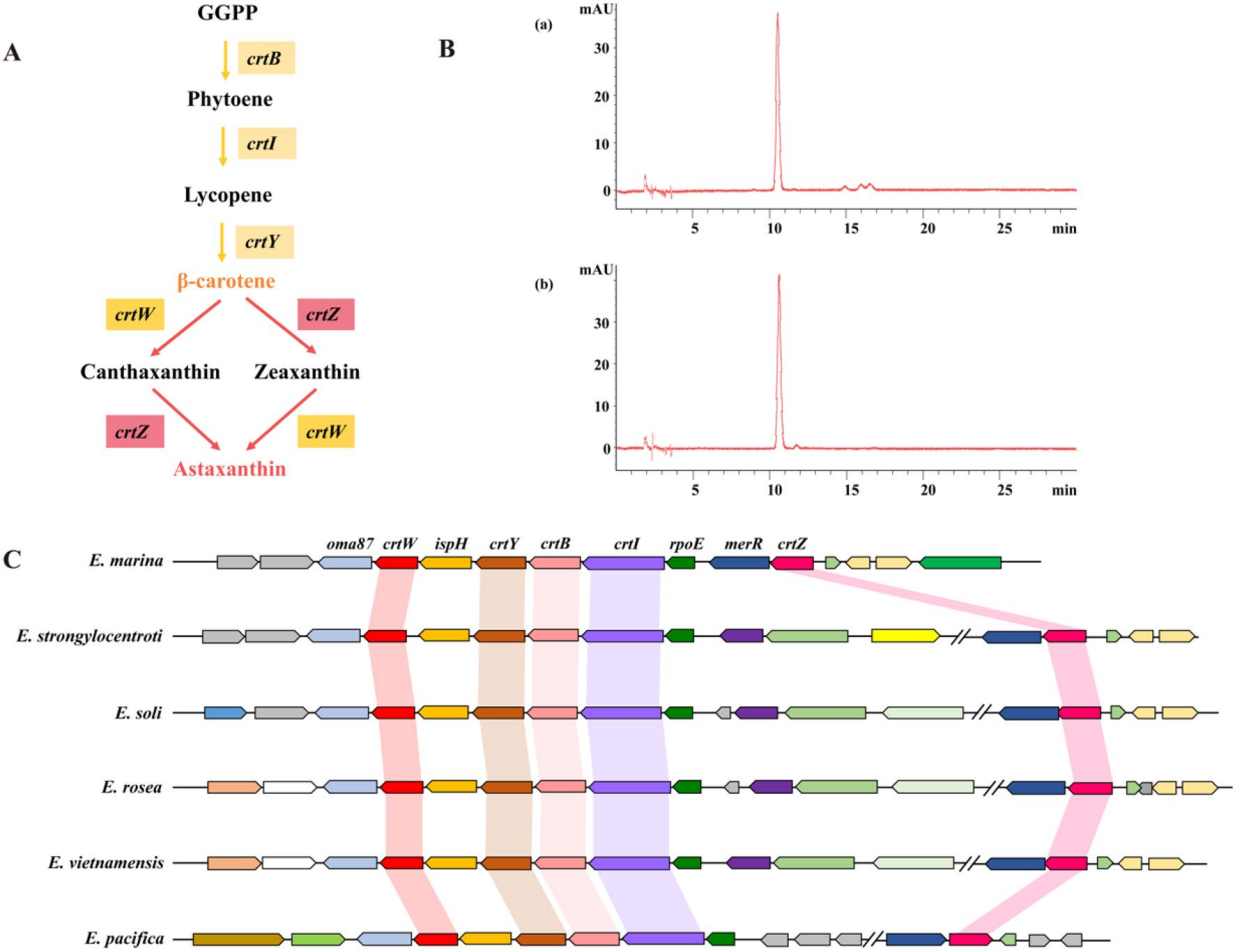
Carotenoids Biosynthesis analysis of *Echinicola marina* strain. **(A)** Proposed pathway of carotenoid biosynthesis in *E. marina* SCS 3–6. **(B)** High-pressure liquid chromatography spectra of carotenoids extracted from strain SCS 3–6 (a) and the standard of astaxanthin (b). UV detection was performed at 478 nm. **(C)** Carotenoid biosynthesis genes in the *E. marina* compared to similar gene clusters from other bacteria.

Through genome annotation, a gene cluster was shown to be directly involved in the synthesis of carotenoids. Based on these genes, *crtW*, *crtY*, *crtB*, *crtI* and *crtZ* (Fig. 7C), we speculated on the synthetic pathway of carotenoid production of *E. marina* SCS 3–6 and the production may be astaxanthin (Fig. 7A). Geranylgeranyl diphosphate (GGPP) is the direct precursor for carotenoid biosynthesis^48^. Phytoene is the first carotenoid formed in the bacterial carotenoid biosynthesis pathway and it is formed from the condensation of two molecules of GGPP^49^ by phytoene synthase (CrtB). Second, the four desaturation steps from phytoene to lycopene were mediated by a single enzyme, CrtI^50^. Following synthesis of lycopene, a large variety of carotenoids were produced by different lycopene cyclase processes. We found a gene of the strain genome was annotated as lycopene cyclase (CrtY). Thus, lycopene was converted to β-carotene by lycopene cyclase. The final synthesis of astaxanthin from β-carotene is a metabolic web containing several branches, according to the different participation steps and orders of β-carotene ketolase (CrtW) and β-carotene hydroxylase (CrtZ)^51^. Therefore, based on the presence of the *crt* gene in the genome, we speculated that the synthetic carotenoid may be astaxanthin. Further, the standard of astaxanthin and carotenoids extracted from the strain SCS 3–6 were analyzed by high-pressure liquid chromatography (HPLC), and the results supported the speculation (Fig. 7B).

*Echinicola* strains are usually orange, pink or yellow due to the presence of carotenoids pigments. In *E. marina* SCS 3–6 the pink pigment is cell bound and was extracted using organic solvents. To investigate the evolution of *crt* gene cluster, we predicted and performed a comparison of the carotenoid synthetic clusters in SCS 3–6 with other related strains (Fig. 7C). Carotenoid biosynthesis gene clusters have been found in *Echinicola* strains. In the genome of *E. marina* SCS 3–6, the cluster contained five carotenogenic genes (*crtW*, *crtZ*, *crtY*, *crtB*, and *crtI*) with the same orientation, one stress-responsive sigma factor gene (*rpoE*), one gene (*merR*) encoding transcriptional regulator, one gene (*ispH*) encoding an enzyme in the methyl erythritol phosphate pathway of isoprenoid synthesis, and one gene (*oma87*) encoding an outer membrane protein. Based on the comparative carotenoid synthetic gene cluster analysis, five genes encoding carotenogenic enzymes were in single gene cluster of *E. marina*, but the carotenoid biosynthesis gene clusters of five other available *Echinicola* strains contained two gene clusters that four carotenogenic genes (*crtW*, *crtY*, *crtB* and *crtI*) were found in one gene cluster and *crtZ* gene encoding β-carotene hydroxylase was in the other gene cluster. In *E. pacifica*, the *crtZ* gene is essentially required for astaxanthin synthesis was located in opposite orientation. The organization of *rpoE*–*crtI*–*crtB*–*crtY*–*ispH*–*crtW*–*oma87* was identified and conserved in *Echinicola*. Of the *E. marina* enzymes, β-carotene hydroxylase (CrtZ) has 67%, 65%, 68%, 66% and 63% amino acid identities from *E. strogylocentroti*, *E. soli*, *E. rosea*, *E. vietnamensis* and *E. pacifica*, respectively. And β-carotene ketolase (CrtW) shows 72%, 71%, 73%, 67% and 67% amino acid identities from *E. strogylocentroti*, *E. soli*, *E. rosea*, *E. vietnamensis* and *E. pacifica*, respectively. Carotenoid accumulation in genus *Echinicola* stabilizes cell membranes to enable survival in various hypersaline environments. From the hypothesis of evolution and function analysis, gene deletion and genomic rearrangement caused the formation of one carotenoid synthesis gene cluster in *E. marina* for regulation of *crt* gene cluster and astaxanthin production.

## Conclusion

Based on the results of phenotypic and genotypic analyses, strain SCS 3–6 is a novel species belongs to the genus *Echinicola*. SCS 3–6 was distinguished from closely related *Echinicola* species according to the phylogenetic tree constructed using 16S rRNA gene sequences and core genome sequences. We firstly research the relationships between members of the genus *Echinicola*. Analysis of the complete genome of SCS 3–6 indicated its polysaccharides degradation ability and carotenoid production ability.

### Description of *Echinicola marina* sp. nov.

*Echinicola marina* (ma.ri’na L. fem. adj. marina, of the sea, marine).

*Echinicola marina* SCS 3–6 is facultative anaerobes, Gram-stain negative, non-motile by gliding. Cells are rod-shaped, and they have a width between 0.2 and 0.3 μm and a length between 1.5 and 2.0 μm. Colonies of this strain were circular, convex and pink pigmented after 2–3 days of incubation 30 °C on marine agar. Strain SCS 3–6 has exhibited positive catalase, oxidase and the hydrolysis of gelatin and casein, but negative for starch and tween 80. Strain SCS 3–6 can use various carbon sources: d-maltose, d-trehalose, d-cellobiose, gentiobiose, sucrose, d-turanose, α-d-lactose, d-melibiose, d-salicin, *N*-acetyl neuraminic, α-d-glucose, d-mannose, d-fructose and d-galactose.

The major cellular fatty acids are iso-C_15:0_ and Summed Feature 3 (C_16:1_ ω7c and/or C_16:1_ ω6c). The GenBank accession number for the 16S rRNA gene sequence of the type strain SCS 3-6^T^ is MZ567047. The genome sequence of the type strain SCS 3-6^T^ has been deposited in GenBank/EMBL/DDBJ under the accession number CP080025. The genome of *Echinicola marina* is 5,693,670 bp long and exhibits a G + C% content 40.11 mol%. The type strain is SCS 3-6^T^ (= GDMCC 1.2220^T^ = JCM 34403^T^).

## Supplementary Information

Supplementary Information.

## References

[1] Kanzy HM, Nasr N, El-Shazly HA, Barakat OS (2015). Optimization of carotenoids production by yeast strains of *Rhodotorula* using salted cheese whey. Int. J. Curr. Microbiol. App. Sci..

[2] Saini RK, Keum Y-S (2017). Progress in microbial carotenoids production. Indian J. Med. Microbiol..

[3] Langi P, Kiokias S, Varzakas T, Proestos C (2018). Carotenoids: From plants to food and feed industries.. Microb. Carotenoids..

[4] Brotosudarmo THP, Limantara L, Setiyono ET (2020). Structures of astaxanthin and their consequences for therapeutic application. J. Food Sci..

[5] Galasso C, Corinaldesi C, Sansone C (2017). Carotenoids from marine organisms: Biological functions and industrial applications. Antioxidants.

[6] Ambati RR, Phang S-M, Ravi S, Aswathanarayana RG (2014). Astaxanthin: Sources, extraction, stability, biological activities and its commercial applications—A review. Mar. Drugs.

[7] Giannaccare G (2020). Clinical applications of astaxanthin in the treatment of ocular diseases: Emerging insights. Mar. Drugs.

[8] Davinelli S, Nielsen ME, Scapagnini G (2018). Astaxanthin in skin health, repair, and disease: A comprehensive review. Nutrients.

[9] Brendler T, Williamson EM (2019). Astaxanthin: How much is too much? A safety review. Phytother. Res..

[10] Nedashkovskaya OI (2006). *Echinicola pacifica* gen. nov., sp. nov., a novel flexibacterium isolated from the sea urchin *Strongylocentrotus intermedius*. Int. J. Syst. Evol. Microbiol..

[11] Nedashkovskaya OI (2007). *Echinicola vietnamensis* sp. nov., a member of the phylum Bacteroidetes isolated from seawater. Int. J. Syst. Evol. Microbiol..

[12] Kim H, Joung Y, Ahn T-S, Joh K (2011). *Echinicola jeungdonensis* sp. nov., isolated from a solar saltern. Int. J. Syst. Evol. Microbiol..

[13] Liang P (2016). *Echinicola rosea* sp. Nov., a marine bacterium isolated from surface seawater. Int. J. Syst. Evol. Microbiol..

[14] Lee DW (2017). *Echinicola sediminis* sp. nov., a marine bacterium isolated from coastal sediment. Int. J. Syst. Evol. Microbiol..

[15] Jung Y-J, Yang S-H, Kwon KK, Bae SS (2017). *Echinicola strongylocentroti* sp. nov., isolated from a sea urchin *Strongylocentrotus intermedius*. Int. J. Syst. Evol. Microbiol..

[16] Xing Y-T (2020). *Echinicola soli* sp. nov., isolated from alkaline saline soil. Int. J. Syst. Evol. Microbiol..

[17] Baek J, Weerawongwiwat V, Kim JH, Yoon JH, Lee JS, Sukhoom A, Kim W (2021). *Echinicola arenosa* sp. nov., isolated from marine sand. Arch. Microbiol..

[18] Zhao H, Song CY, Yin R, Yi YJ, Yun ST, Li YX, Zhou YX (2021). *Echinicola salinicaeni* sp. nov., a novel bacterium isolated from saltern mud. Anton Leeuw Int. J. G..

[19] Srinivas T, Tryambak BK, Kumar PA (2012). *Echinicola shivajiensis* sp. nov., a novel bacterium of the family “Cyclobacteriaceae” isolated from brackish water pond. Anton Leeuw Int. J. G..

[20] Fernández-Gómez B, Richter M, Schüler M, Pinhassi J, Acinas SG, González JM, Pedrós-Alió C (2013). Ecology of marine Bacteroidetes: a comparative genomics approach. ISME J..

[21] Hahnke RL (2016). Genome-based taxonomic classification of Bacteroidetes. Front. Microbiol..

[22] Zhan P (2020). Complete genome sequence of *Echinicola rosea* JL3085, a xylan and pectin decomposer. Mar. Genomics.

[23] He J, Liu L, Liu X, Tang K (2020). Isolation and characterization of a novel cold-active, halotolerant endoxylanase from *Echinicola rosea* sp. Nov. JL3085T. Mar. Drugs.

[24] Tomshich SV, Kokoulin MS, Kalinovsky AI, Komandrova NA, Ol’ga IN (2015). Structure of the O-specific polysaccharide from a marine bacterium *Echinicola vietnamensis* KMM 6221T. Carbohydr. Res..

[25] Tomshich SV, Kokoulin MS, Kalinovsky AI, Ol'ga IN, Komandrova NA (2016). Structure of the O-specific polysaccharide from a marine bacterium *Echinicola pacifica* КMM 6172T containing 2, 3-diacetamido-2, 3-dideoxy-d-glucuronic acid. Carbohydr. Res..

[26] Buck JD (1982). Nonstaining (KOH) method for determination of gram reactions of marine bacteria. Appl. Environ. Microbiol..

[27] Liu Y, Du J, Zhang J, Lai Q, Shao Z, Zhu H (2020). *Devosia marina* sp. nov., isolated from deep seawater of the South China Sea, and reclassification of *Devosia subaequoris* as a later heterotypic synonym of *Devosia soli*. Int. J. Syst. Evol. Microbiol..

[28] Collins, M. D. *Methods in Microbiology.* (ed. Bergan, T.). Vol. 18. 329–366. (Academic Press, 1985).

[29] Minnikin DE (1984). An integrated procedure for the extraction of bacterial isoprenoid quinones and polar lipids. J. Microbiol. Methods.

[30] Dawyndt P, Vancanneyt M, Snauwaert C, De Baets B, De Meyer H, Swings J (2006). Mining fatty acid databases for detection of novel compounds in aerobic bacteria. J. Microbiol. Methods.

[31] Weisburg WG, Barns SM, Pelletier DA, Lane DJ (1991). 16S ribosomal DNA amplification for phylogenetic study. J. Bacteriol..

[32] Yoon S-H (2017). Introducing EzBioCloud: A taxonomically united database of 16S rRNA gene sequences and whole-genome assemblies. Int. J. Syst. Evol. Microbiol..

[33] Tamura K, Stecher G, Kumar SA-O (2021). MEGA11: Molecular evolutionary genetics analysis version 11. Mol. Biol. Evol..

[34] Hillis DM, Bull JJ (1993). An empirical test of bootstrapping as a method for assessing confidence in phylogenetic analysis. Syst. Biol..

[35] Luo R (2015). SOAPdenovo2: An empirically improved memory-efficient short-read de novo assembler. GigaScience.

[36] Koren S (2017). Canu: Scalable and accurate long-read assembly via adaptive k-mer weighting and repeat separation. Genome Res..

[37] DeJesus MA, Sacchettini JC, Ioerger TR (2013). Reannotation of translational start sites in the genome of *Mycobacterium tuberculosis*. Tuberculosis.

[38] Kanehisa M, Sato Y (2019). KEGG mapper for inferring cellular functions from protein sequences. Protein Sci..

[39] Feng S, Jian Y, Jin L, Tang S, Li Z (2021). Complete genome sequence data of rare actinomycetes strain *Saccharothrix texasensis* 6-C, a biological control agent for potato late blight. Mol. Plant Microbe Interact..

[40] Blin K (2019). antiSMASH 5.0: Updates to the secondary metabolite genome mining pipeline. Nucleic Acids Res..

[41] Richter M, Rosselló-Móra R (2009). Shifting the genomic gold standard for the prokaryotic species definition. Proc. Natl. Acad. Sci. U S A.

[42] Meier-Kolthoff JP, Klenk H-P, Göker M (2014). Taxonomic use of DNA G+ C content and DNA–DNA hybridization in the genomic age. Int. J. Syst. Evol. Microbiol..

[43] Chaudhari NM, Gupta VK, Dutta C (2016). BPGA-an ultra-fast pan-genome analysis pipeline. Sci. Rep..

[44] Krzywinski M (2009). Circos: An information aesthetic for comparative genomics. Genome Res.

[45] Chun J (2018). Proposed minimal standards for the use of genome data for the taxonomy of prokaryotes. Int. J. Syst. Evol. Microbiol..

[46] Medini D, Donati C, Tettelin H, Masignani V, Rappuoli R (2005). The microbial pan-genome. Curr. Opin. Genet. Dev..

[47] Tang K, Lin Y, Han Y, Jiao N (2017). Characterization of potential polysaccharide utilization systems in the marine Bacteroidetes *Gramella flava* JLT2011 using a multi-omics approach. Front. Microbiol..

[48] Sun, T., Tadmor, Y. & Li, L. *Plant and Food Carotenoids*. 3–23. (Springer, 2020).

[49] Armstrong GA, Alberti M, Hearst JE (1990). Conserved enzymes mediate the early reactions of carotenoid biosynthesis in nonphotosynthetic and photosynthetic prokaryotes. Proc. Natl. Acad. Sci. U.S.A..

[50] Linden H (1991). Functional complementation in *Escherichia coli* of different phytoene desaturase genes and analysis of accumulated carotenes. Z. Nat. C.

[51] Martín JF, Gudiña E, Barredo JL (2008). Conversion of β-carotene into astaxanthin: Two separate enzymes or a bifunctional hydroxylase-ketolase protein?. Microb. Cell Factories.

