# Supplementary material for: Comparative genome characterization of Echinicola marina sp. nov., isolated from deep-sea sediment provide insight into carotenoid biosynthetic gene cluster evolution
Source: Sci Rep. 2021 Dec 17;11:24188. doi: 10.1038/s41598-021-03683-0 (PMC8683446; doi:10.1038/s41598-021-03683-0)
Supplement: Supplementary file 1 — Supplementary Information. [file 41598_2021_3683_MOESM1_ESM.docx]

Comparative genome characterization of *Echinicola marina* sp. nov., isolated from deep-sea sediment provide insight into carotenoid biosynthetic gene cluster evolution

Yu Pang^1^, Mengru Chen^1^, Wei Lu, Ming Chen, Yongliang Yan, Min Lin, Wei Zhang*, Zhengfu Zhou*

Biotechnology Research Institute, Chinese Academy of Agricultural Sciences, Beijing 100081, China;

^1^ These authors contributed equally: Yu Pang, and Mengru Chen

* Corresponding author *E-mail:* zhouzhengfu@caas.cn (Z. Zhou), zhangwei01@caas.cn (W. Zhang).


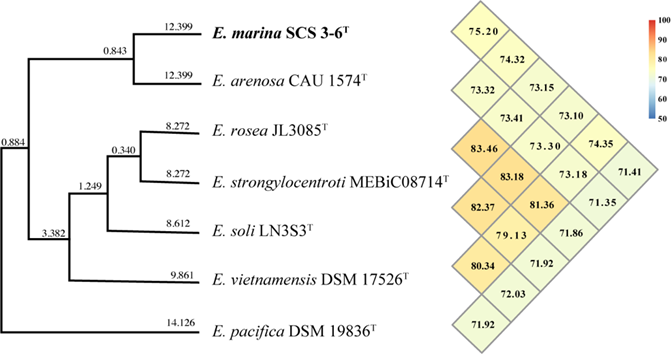


**Figure S1 Genomic comparison of bacterial species in the genus Echinicola.** Unweight pair group method with arithmetic mean (UPGMA) dendrogram based on the OrthoANI values of seven species in the genus *Echinicola*. The numbers on the branches indicate the branch length and the numbers in the heat-map indicate the OrthoANI values between two genomes. OrthoANI (<https://www.ezbiocloud.net/tools/orthoani>).

## Functional categories of *Echinicola* *marina* SCS 3-6.

Of the 5053 CDSs, a total of 3969 CDSs (78.55%) were specifically assigned to clusters of COG families comprising 20 functional categories (Figure S2), of which function unknown (1600 genes) was the most common term. In addition, other genes were mainly classified into function categories for inorganic ion transport and metabolism (P, 302 genes); cell wall/membrane/envelope biogenesis (M, 257 genes); carbohydrate transport and metabolism (G, 240 genes); amino acid transport and metabolism (E, 223 genes); and replication, recombination and repair (L, 214 genes). Furthermore, a total of 2714 CDSs were categorized into three categories according to matches with known sequences by using GO analysis. In three categories, molecular function contained most numbers gene number (2217), followed by biological process (1912 genes) and cellular component (1196 genes) (Figure S3). In molecular function, the most five pathways were DNA binding (GO: 0003677; 263 genes), ATP binding (GO: 0005524; 246 genes), metal ion binding (GO: 0046872; 127 genes), hydrolase activity (GO: 0016787; 106 genes) and transcription factor activity, sequence-specific DNA binding (GO: 0003700; 93 genes). Oxidation-reduction process (GO: 0055114; 260 genes) and regulation of transcription, DNA-templated (GO: 0006355; 142 genes) were most pathways in biological process, and component of membrane (GO: 0016021; 596genes) and cytoplasm (GO: 0005737; 221 genes) were most pathways in cellular component. Among the predicted genes, 1636 (32.38%) were successfully assigned to KEGG pathways. This analysis revealed 40 functional groups, among which the genes participate in metabolism (Figure S4), mainly functioning in the biosynthesis of amino acid (ko: 01230; 117 genes), carbon metabolism (ko: 01200; 92 genes), purine metabolism (ko: 00230; 54genes), ribosome (ko03010; 50 genes), pyrimidine metabolism (ko: 00240; 48genes), and amino sugar and nucleotide sugar metabolism (ko00520; 48 genes). It has been proposed that Marine *Bacteroidetes* have adapted to their niche with a specialized set of metabolic and have the capacity to degrade polymers.


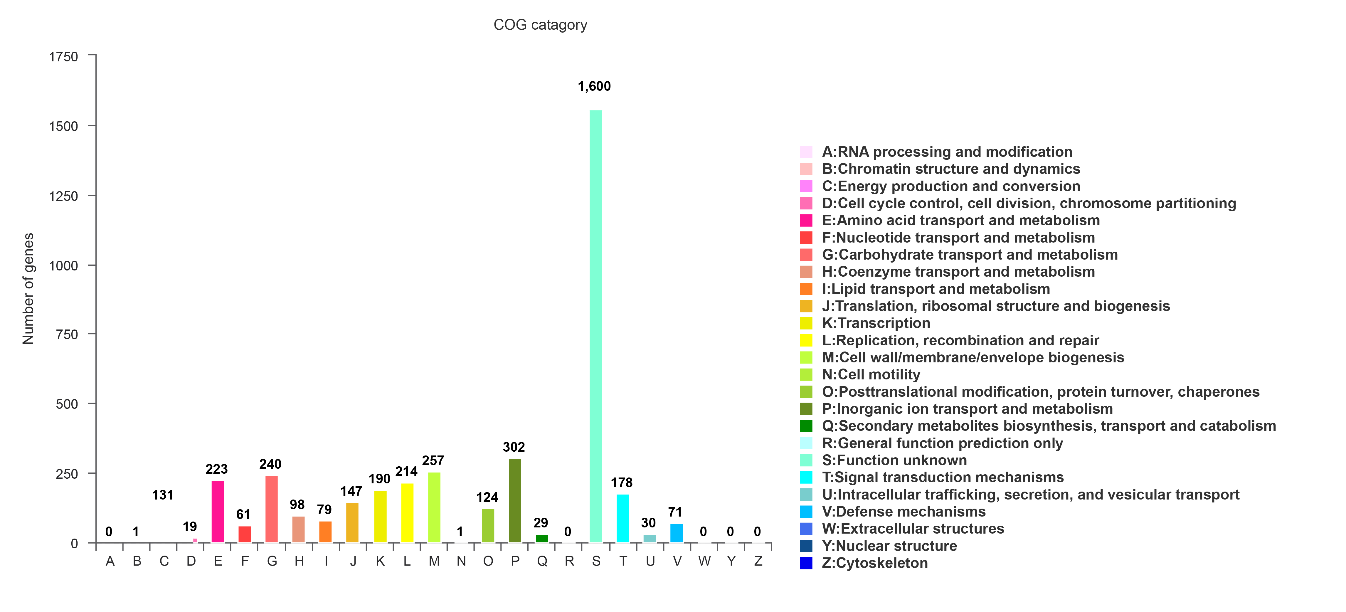


**Figure S2. Clusters of Orthologous Groups of proteins (COG) annotation f Echinicola marina SCS 3-6.**


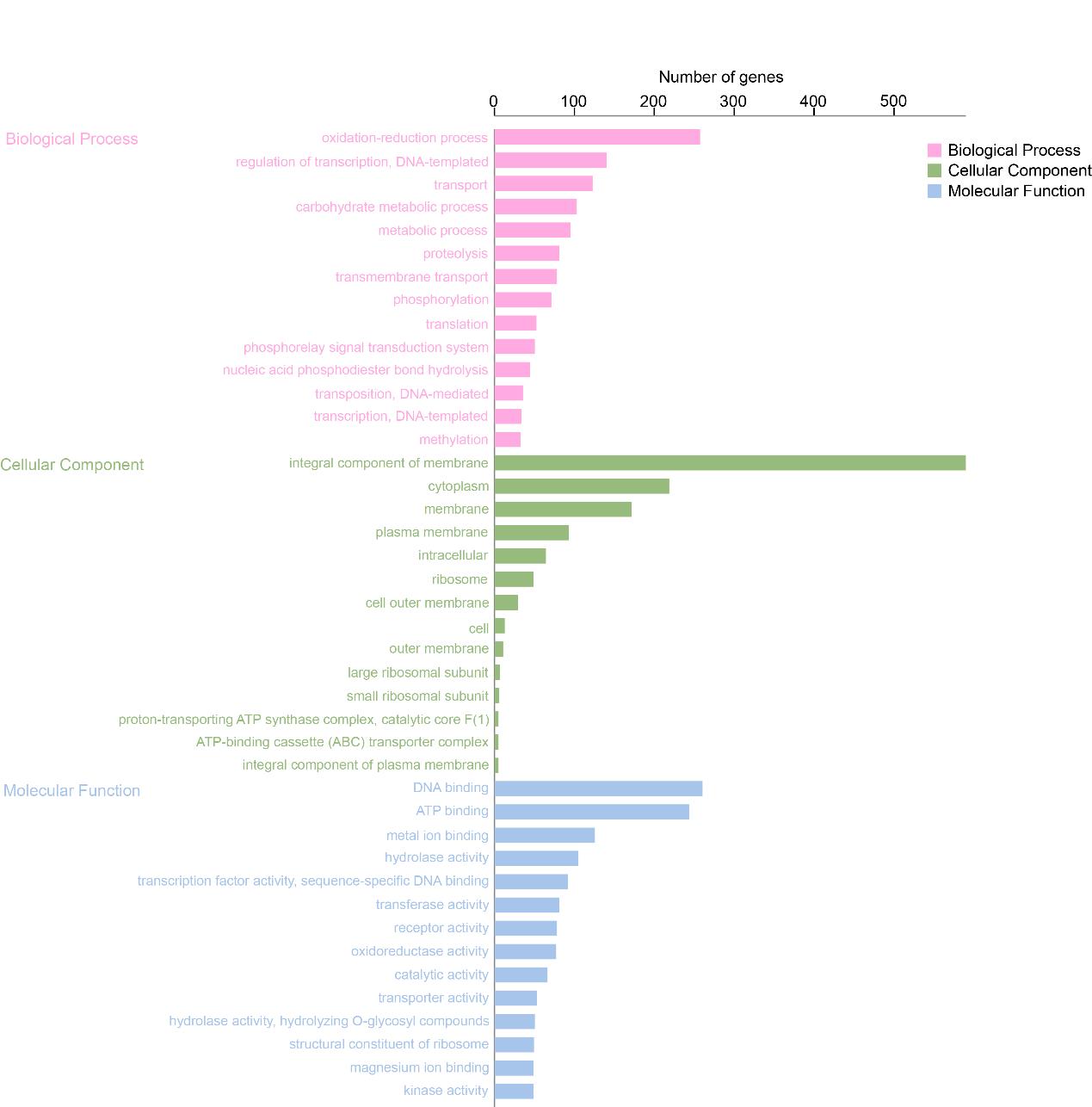


**Figure S3. Clusters of Gene Ontology (GO) annotation of Echinicola marina SCS 3-6.**


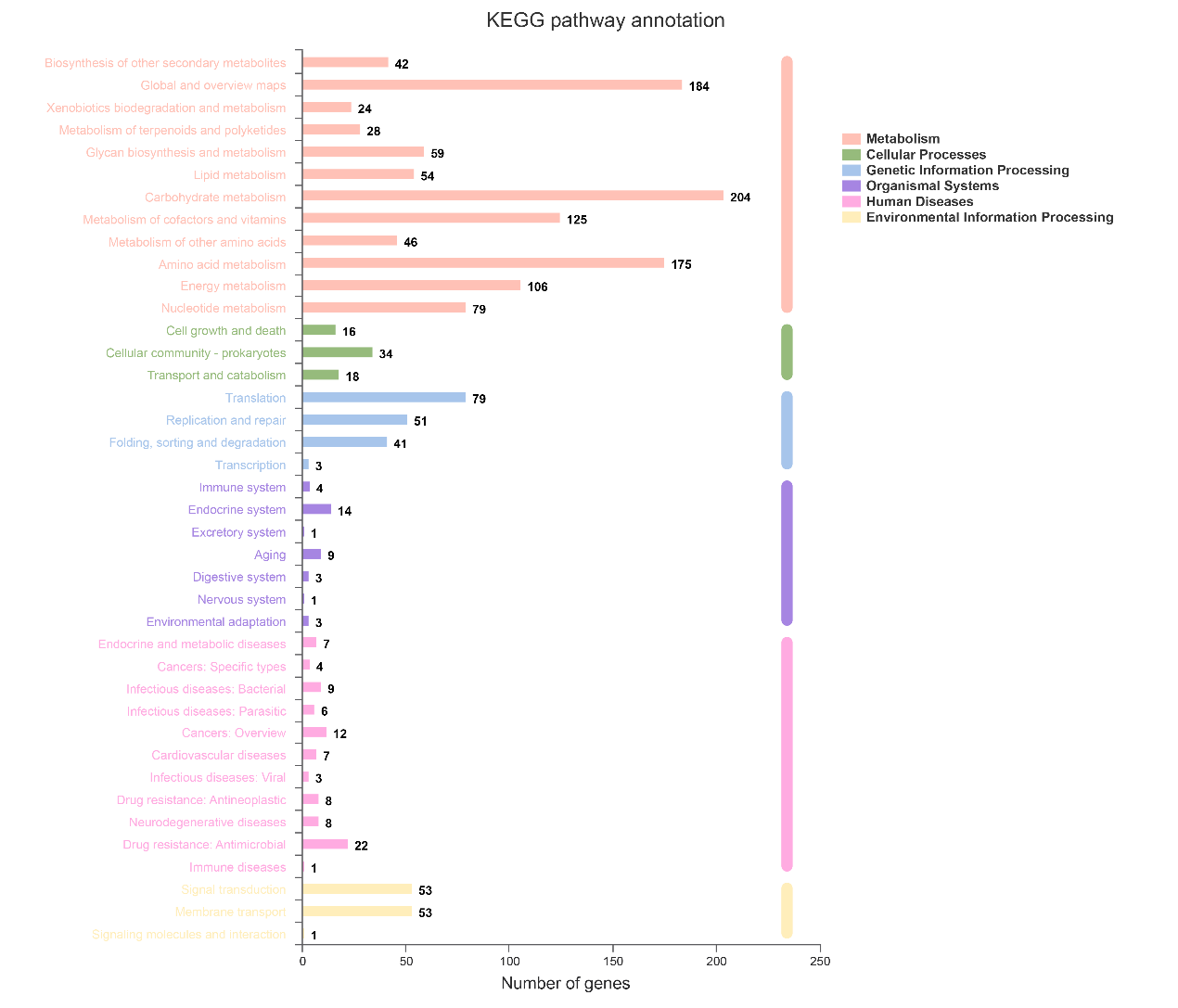


**Figure S4. Clusters of KEGG annotation of Echinicola marina SCS 3-6.**

**Table S1. The fatty acid profiles of strain SCS 3-6 and its relatives.**

1, *E. marina* SCS 3-6; 2, *E. shivajiensis* AK12^T^; 3, E. *sediminis* 001-Na2^T^.

|  | 1 | 2 | 3 |
| --- | --- | --- | --- |
| iso-C_13:0_ | 0.43 | 0.78 | 0.92 |
| C_13:1_ at 12-13 | 0.25 | 0.20 | --- |
| C_14:0_ | 0.29 | 0.58 | 0.92 |
| C_15:1_ iso G | 1.11 | 0.68 | --- |
| iso-C_15:0_ | 29.11 | 29.74 | 31.81 |
| anteiso-C_15:0_ | 1.08 | 1.08 | 2.09 |
| C_15:1_ w6c | 2.84 | 2.52 | 2.73 |
| C_15:0_ | 2.63 | 3.00 | 13.36 |
| C_16:1_ w7c alcohol | 0.34 | --- | --- |
| iso-C_16:1_ H | 0.70 | 0.93 | --- |
| iso-C_16:0_ | 0.97 | 1.20 | 2.28 |
| C_16:1_ w5c | 2.32 | 1.65 | 6.09 |
| C_16:0_ | 1.79 | 3.47 | 3.34 |
| iso-C_15:0_ 3-OH | 3.11 | 3.76 | 2.96 |
| C_15:0_ 2-OH | 0.23 | 0.57 | --- |
| anteiso-C_17:1_ w9c | --- | --- | 0.82 |
| iso-C_17:0_ | 1.77 | 1.09 | 1.19 |
| anteiso-C_17:0_ | 0.14 | 0.25 | --- |
| C_17:1_ w8c | 0.54 | 0.63 | 1.13 |
| C_17:1_ w6c | 9.49 | 7.34 | 3.05 |
| C_17:0_ | 0.29 | 0.34 | --- |
| iso-C_16:0_ 3-OH | 0.42 | 0.38 | --- |
| iso-C_18:1_ H | --- | 0.20 | --- |
| C_16:0_ 3-OH | 0.66 | 1.69 | 1.45 |
| C_18:1_ w9c | 0.44 | 0.74 | 1.40 |
| C_18:0_ | 0.85 | 2.73 | 3.49 |
| iso-C_17:0_ 3-OH | 9.82 | 8.93 | 8.46 |
| C_17:0_ 2-OH | 0.35 | 0.32 | --- |
| C_17:0_ 3-OH | ---- | 0.54 | --- |
| iso-C_19:0_ | --- | 0.24 | --- |
| Summed Feature 1 | 0.43 | 0.36 | --- |
| Summed Feature 3 | 19.31 | 16.35 | 9.75 |
| Summed Feature 8 | 0.18 | 0.60 | --- |
| Summed Feature 9 | 8.08 | 7.10 | 2.74 |

**Table S2. Genome features of strain SCS 3-6**

| Properties | Value |
| --- | --- |
| Genome size (bp) | 5693670 |
| G + C content (%) | \|  \| 40.11 \| \| --- \| --- \| |
| DNA coding region (bp) | \|  \| 4993956 \| \| --- \| --- \| |
| Protein-coding genes (CDSs) | 5053 |
| GC content in gene region (%) | 40.99 |
| GC content in intergenetic region (%) | 33.87 |
| 5S rRNA | 5 |
| 16S rRNA | 5 |
| 23S rRNA | 5 |
| tRNA | 41 |
| Repeat DNA | 52 |
| Genes assigned to NR | 5053 |
| Genes assigned to Swiss-Prot | 2713 |
| Genes assigned to Pfam | 3576 |
| Genes assigned to COG | 3969 |
| Genes assigned to GO | 2714 |
| Genes assigned to KEGG | 1636 |
| Genes assigned to CAZy | 299 |
